# The Plasticity of Immune Cell Response Complicates Dissecting the Underlying Pathology of Multiple Sclerosis

**DOI:** 10.1155/2024/5383099

**Published:** 2024-01-04

**Authors:** Sujan Kumar Sarkar, Annie M. L. Willson, Margaret A. Jordan

**Affiliations:** ^1^Department of Anatomy, Histology and Physiology, Faculty of Animal Science and Veterinary Medicine, Sher-e-Bangla Agricultural University, Dhaka, Bangladesh; ^2^Biomedical Sciences and Molecular Biology, CPHMVS, James Cook University, Townsville, Queensland 4811, Australia

## Abstract

Multiple sclerosis (MS) is a neurodegenerative autoimmune disease characterized by the destruction of the myelin sheath of the neuronal axon in the central nervous system. Many risk factors, including environmental, epigenetic, genetic, and lifestyle factors, are responsible for the development of MS. It has long been thought that only adaptive immune cells, especially autoreactive T cells, are responsible for the pathophysiology; however, recent evidence has indicated that innate immune cells are also highly involved in disease initiation and progression. Here, we compile the available data regarding the role immune cells play in MS, drawn from both human and animal research. While T and B lymphocytes, chiefly enhance MS pathology, regulatory T cells (Tregs) may serve a more protective role, as can B cells, depending on context and location. Cells chiefly involved in innate immunity, including macrophages, microglia, astrocytes, dendritic cells, natural killer (NK) cells, eosinophils, and mast cells, play varied roles. In addition, there is evidence regarding the involvement of innate-like immune cells, such as *γδ* T cells, NKT cells, MAIT cells, and innate-like B cells as crucial contributors to MS pathophysiology. It is unclear which of these cell subsets are involved in the onset or progression of disease or in protective mechanisms due to their plastic nature, which can change their properties and functions depending on microenvironmental exposure and the response of neural networks in damage control. This highlights the need for a multipronged approach, combining stringently designed clinical data with carefully controlled in vitro and in vivo research findings, to identify the underlying mechanisms so that more effective therapeutics can be developed.

## 1. Introduction

Multiple sclerosis (MS) is a chronic neuroinflammatory and demyelinating autoimmune disorder of the central nervous system (CNS) [1, 2]. Diagnosis generally occurs between the ages of 20–50 with a female : male bias of 3 : 1 [3]. It is the leading cause in Western countries of lifelong disability in young adults through the loss of neurological functions [1, 4], and the frequency has been rising in recent years [5, 6]. It is characterized by severe axonal injury, loss of myelin sheath, and long-term degeneration of neurons mediated by a self-reactive immune response [7]. Clinical features vary depending on where brain lesions occur [8], often resulting in visual impairment, muscle fatigue, abnormal sensation, and lack of muscle coordination [9]. Different forms of MS have been described; with relapsing–remitting MS (RRMS) characterized by periods of worsening symptoms, followed by periods of complete recovery [10], occurring in ∼85% of cases. Within 5–15 years of initial diagnosis, around 65% of patients [8] will develop a more progressive form of the disease, termed secondary-progressive MS (SPMS) [11], where periods of relapse are more frequent and of longer duration. Primary-progressive MS (PPMS) is characterized by worsening symptoms and progression of the disease from the onset, affecting around 15% of individuals diagnosed.

Impairment from MS occurs through one of two means: progression independent of “clinical” relapse activity (PIRA) or relapse-associated worsening (RAW) [12]. Although both mechanisms are capable of causing permanent impairment at any phase of the disease [13–16], PIRA appears to be the main contributor to disability formation in those who progress with a relapsing–remitting form of MS, independent of treatment efficacy. A combination of pathological processes contribute to PIRA, including paramagnetic rim lesions (PRL's, previously termed chronic active lesions (CALs)) and slowly expanding lesions (SELs), the presence of meningeal lymphoid aggregates and diffuse glial cell activation with the resultant neuronal–axonal damage [17, 18]. To date, it is unknown what the pathological drivers of PIRA are or whether the same drivers of early MS are responsible for SPMS [13, 15, 16]. Age advancement and the amount of time since the initial diagnosis are the primary risk factors for cumulative disability [12]. More MS subtypes have now been accepted to try and increase diagnostic accuracy, characterize the unique immunopathogenic features, and customize medical care. The understanding of early demyelination events, known as CISs or RISs (clinically or radiologically isolated syndromes, respectively), transverse myelitis, neuromyelitis optica (NMO and NMO spectrum of diseases), recurrent isolated optic neuritis, tumefactive demyelination, and acute disseminated encephalomyelitis (ADEM) along with its variations (acute hemorrhagic leukoencephalitis-AHL, Marburg variant, and Balo's concentric sclerosis) are among them [19]. CIS primarily affects young people, usually presenting as acute (85% of cases) or subacute onset bouts affecting the brainstem, optic neurons, or spinal cord [20] with a peak intensity achieved within 2–3 weeks. According to the epidemiological data gathered by the MS International Federation in 2020, MS affects more than 2.8 million people globally [21], of which at least 1 million have a progressive form of the disease, and the incidence in areas with the highest prevalence is as high as 1/300 individuals.

## 2. The Aetiology of MS

Although the aetiology of MS has yet to be established, it is considered a complex autoimmune disease triggered by an aberrant immune reaction [1] likely modulated by epigenetic and environmental risk factors, including tobacco smoking [22], obesity [14], hormones such as estrogen and progesterone [23] and those involved in circadian rhythm like melatonin [24], bacterial and viral infections (particular Epstein–Barr virus) [25, 26], geographical location, i.e., low sunlight and vitamin D [27, 28], in genetically predisposed hosts [14]. First-degree relatives of an affected individual, sharing both genetics and a common environment, have a 12%–20% increased risk of developing MS, ∼33% in identical twins [29]. The advent of genome-wide association studies has catapulted the discovery of disease-associated genes, identifying 32 variants within the extended major histocompatibility complex (MHC), 200 autosomal genes outside of the MHC (mostly proximal to or within immune genes) and a variant on the X-chromosome (in a T-cell associated region) [30]. The presence of the human leukocyte antigen (HLA)-DRB1 ^*∗*^1501, DQA1 ^*∗*^0102, and DQB1 ^*∗*^0602 alleles convey the highest risk of developing MS [31].

**Figure 1:**
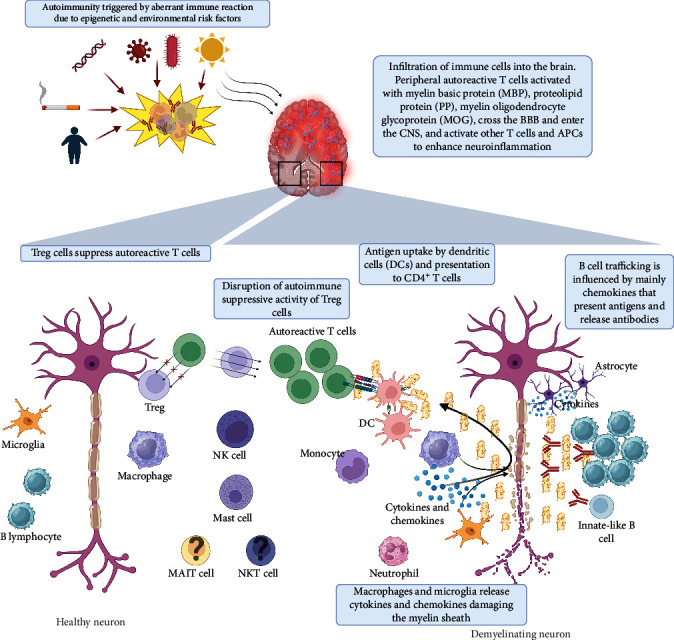
The role of immune cells in multiple sclerosis. An autoinflammatory reaction is triggered by an aberrant immune reaction due to epigenetic and environmental risk factors in genetically predisposed individuals. There is infiltration of immune cells into the brain (peripheral autoreactive T cells activated with myelin basic protein (MBP), proteolipid protein (PP), myelin oligodendrocyte glycoprotein (MOG), cross the blood–brain barrier (BBB) and enter the central nervous system (CNS), infiltrating other T cells, and APCs to enhance neuroinflammation). In healthy brain, Treg cells suppress autoreactive T cells, but under autoimmune conditions, the autoimmune suppressive activity of Treg cell is disrupted. B cell trafficking is influenced by many chemokines, including CXCL13, and they present antigens and release antibodies. Macrophages and microglia release cytokines and chemokines damaging the myelin sheath; while there is antigen uptake by dendritic cells (DCs) and CD4 T cell/DC interactions, and the interaction of several other immune cells, resulting in demyelination of the neuron (created with https://BioRender.com).

Disease mechanism is slowly being realized through studies of individuals affected with MS and through rodent models of the disease. Inflammatory cells must be activated for MS to develop [32] and although the underlying cause is still elusive, the inflammatory hallmark of MS is lymphocyte deposition in the CNS and the cerebrospinal fluid (CSF) [33]. Until recently, it was also thought that MS was predominantly CD4^+^ T cell-mediated [1], but research indicates the involvement of B cells and other T cell subsets [34] and a role for cells chiefly involved in innate reactions (Figure 1). Despite the CNS's previous designation as an immune-privileged site, more recent mouse studies have also revealed the existence of resident professional antigen-presenting cells (APCs) in the brain [35] and a fully functional lymphatic system within the meninges that drains into deep cervical lymph nodes (LNs) [36], where CNS antigens have been demonstrated to elicit an immune response [37]. While inflammatory responses are essential for maintaining CNS homeostasis and accelerating the healing process after injury [38], pathological inflammation or excessive neuroimmune axis activity can disrupt the immunological balance, and result in a primary neuroinflammatory disease like MS [39–41].

Myelin, oligodendrocyte, and axon destruction are pathophysiological features of MS [42]; however, there has been much discussion regarding whether these factors cause or result from the disease, leading to the proposal of two complementary paradigms known as the “outside–in” and “inside–out” [43] theories. The outside–in paradigm postulates that myelin is the target of a peripherally elicited autoimmune attack, while the inside–out paradigm links secondary autoimmune reactions against myelin debris to a fundamental CNS cytodegenerative process [44–46]. The outside–in theory was first put forth by observations in murine experimental autoimmune encephalomyelitis (EAE), where a pathological sequence of events led to CNS infiltration following interaction with resident APCs presenting the locally sampled antigens and CD4 T cell activation [47]. The result was attacks on oligodendrocytes and myelin by humoral and cellular immune factors, such as complement, CD8 T cells, macrophages, and antibodies. According to Nisticò et al. [48], there have been reports of multiple activated immune cells infiltrating white matter and secreting different substances, such as cytokines and chemokines, that alter neuronal function and signal formation in neuronal synapses. This leads to the formation of demyelinated lesions, inflammatory foci, and neural damage of the white (and gray) matter.

Support for the inside–out theory comes from observations of blister-like swellings called myelin blistering forming in normal-appearing white matter (NAWM), which are surrounded and infiltrated by strong immunoreactivity and show signs of myelin protein posttranslational modification (identified by citrullinated protein detecting mAb) [46]. Concurrently, there is a change in the expression of adhesion and tethering proteins that normally mediate stability of the axon–myelin unit and of those involved in the compact binding of inner myelin lamellae to gangliosides on the axon surface, as well as altered myelin polarity and axon swelling [49, 50]. Furthermore, meninges and draining fluids from MS brains have been reported to contain free myelin fragments [51].

Evidence is starting to come together to suggest that both paradigms are at work. It has been suggested that destabilization of the recently discovered axon-myelinic synapse (AMS), which is involved in dynamic communication between axon and myelin, may be the cause of various neurodegenerative diseases, including MS [45]; additionally, mitochondria—which have been implicated in MS before [52]—may play a role in preserving the AMS's stability. Autophagy and complement production can destabilize mitochondria. These processes can occur during a primary immune attack on myelin or as a primary response to a CNS cytodegenerative process, so their presence could support either theory.

## 3. Plasticity

As relapsing–remitting syndrome (RRMS) is characterized by discrete episodes of pronounced neurological symptoms (relapse) and recovery (remission), this suggests that compensatory mechanisms for cumulative damage may exist. Indeed, following demyelination, remyelination—a spontaneous process of regeneration—occurs to restore tissue structure and function as well as give axon support. This process of tissue regeneration involves both innate and adaptive immune responses and is dependent on inflammation, just like it is for other tissue regeneration processes. Because autoinflammation plays a role in disease-causing immune activation, it is challenging to study the underlying process of de- or re-myelination due to the overlapped functions. In addition, although the process appears to work effectively initially, myelin that has been repaired appears to perform less well than myelin that has been freshly synthesized [53]. Furthermore, remyelination loses effectiveness with age and as the disease progresses during the chronic phase.

Another strategy to lessen the clinical impact of damage is neuronal plasticity, which refers to the ability of neural networks to change both structurally and functionally to create new connections, and in some cases, new neurons. While this occurs normally over a person's lifetime to adapt to new experiences, according to Ksiazek-Winiarek et al. [54] and Reddy et al. [55], neural plasticity, demonstrated by fMRI TBS or MRS, is increased in MS patients, specifically in those with RRMS. It is either locally present at the site of injury (synaptic reorganization) or involves distant, uninjured brain regions and pathways. This type of compensatory mechanism, in which molecular, synaptic, and cellular events are reflected in a systems-level reorganization, has also been identified in other types of brain injury. For example, at least two significant cellular regeneration processes take place in stroke patients: immature new neurons migrate into the peri-infarct cortex, and axons in the area form new connections and projection patterns. Growth-promoting genes are successively triggered by neurons, while growth-inhibitory molecules are temporarily decreased. Neurogenesis is the result of immature neurons migrating in waves from the SVZ into the peri-infarct cortex, mediated by cytokines [56]. The brain is not infinitely malleable, however, and damage to areas largely responsible for certain tasks may result in deficits in that area and loss of function. Neuroplastic recovery, too, tends to decline with increasing age and disease duration.

Numerous immune cells exhibit plasticity, a process whereby specialized cell types have the capacity to convert to another cell type to compensate for loss of cellular or systemic function. Mature cells can return to a stem-cell-like state or convert into a different type of mature cell, following a conserved molecular program referred to as paligenosis, which enables cells to adaptively change differentiation state or identity in response to intrinsic and extrinsic signals under both homeostatic and pathological conditions. Macrophages, for example, show evidence of phenotypic shift in the M1/M2 balance such that there is a more pro-inflammatory phenotype during EAE development, compared to control mice [57], while microglia/macrophages in the inflamed CNS during the later stages of EAE are less activated and present as alternatively activated macrophage M2 cells, releasing anti-inflammatory cytokines, which is accompanied by inflammation resolution and tissue repair [58]. This plastic nature of several immune cells complicates the interpretation of observations, without the use of clear identifiers to demarcate the ever-growing cell subpopulations, with their relative percentage, number, and function, which are also dependent on timing and special organization during disease progress or recovery.

## 4. Therapeutics

Early therapeutic intervention has been demonstrated to be critical in preventing irreversible immune-system-related CNS damage, postponing disability, and slowing the disease's natural progression. These immunotherapies, also known as disease-modifying therapies (DMTs), are administered orally, intravenously, or sublingually (Table 1). They work by altering the immune system's activity to reduce the frequency and intensity of CNS attacks as well as the progression of certain diseases. They can function by preventing immune cells from entering the CNS or by obstructing the activation or migration of T or B cells (Table 1). High-dose anti-inflammatory corticosteroids are usually used to treat moderate-to-severe MS relapses, in addition to symptom-focused medication and rehabilitation therapies. Though immunotherapies have made great strides, there is still no convincing evidence from randomized controlled trials that any of these treatments significantly reduces the risk of long-term disability, and many patients experience severe side effects from the treatment (Table 1). Therefore, it is critical to identify the factors that trigger MS and figure out ways to stop it from happening altogether or, at the very least, develop more focused treatments with fewer adverse effects. Given the immune-centered concept of MS pathology, we have tried to conduct a thorough analysis of the information available about immune cells implicated in MS [14, 33] and their potential mechanism of action. As the location is of importance, we have summarized the cell type and action site in Table 2.

## 5. T Cells and B Cells and Their Role in the Development and Progression of MS

The most widely recognized theory for MS initiation is that an autoimmune response is triggered by autoreactive T cells against a component of myelin [14]. It is thought that peripheral autoreactive T cells, (re)activated with a specific neuroantigen, myelin basic protein (MBP), proteolipid protein (PLP), or myelin oligodendrocyte glycoprotein (MOG), cross the blood–brain barrier (BBB) and enter the CNS where they become reactivated with myelin antigen, leading to the infiltration of other T cells and APCs to enhance the neuroinflammatory damage of the CNS [49, 50]. Most research to date has concentrated on CD4^+^ T lymphocytes' role as both facilitators and moderators in MS pathogenesis and as a result, inflammatory CD4^+^ T cell responses are frequently targeted in MS therapy. However, in addition to CD4^+^ T cells, important roles for CD8^+^ T cells and B cells have been uncovered, as has the involvement of regulatory T cell subsets in disease prevention [143].

## 6. T Cells

CD4^+^ T lymphocytes play an important role in the pathophysiology of a mouse model of MS, EAE [144], and the MHC haplotype of different mouse strains is linked to their vulnerability to EAE. These T cells are specific for myelin antigens in EAE, including MBP, PLP, and MOG [145–147], where the adoptive transfer of CD4^+^ T cells from mice inoculated with myelin antigens typically resulted in disease in the recipient. People with MS also exhibited CD4^+^T lymphocytes in their brain lesions [59, 60] and there have been several reports of T cell dysregulation in all subtypes of MS [148], although at least as far as naïve CD4^+^ T cells are concerned, the alteration was particularly evident in those with rapid conversion to SPMS, where the T cell receptor (TCR) and toll-like receptor (TLR) (a costimulatory receptor of TCR-activated cells) signaling pathways were of note [149]. The discovery of HLA-DR15, an MHCII cell surface receptor, as a significant genetic component associated with disease susceptibility established the importance of CD4^+^ T cells as disease effectors in humans [150].

Autoreactive CD4^+^ T cells have been observed in similar or higher numbers in MS patients' peripheral blood compared to healthy individuals [61, 62] and several publications advocate that myelin-specific T cells obtained from MS patients display altered characteristics compared to those detected in healthy donors. These include an enhanced frequency of high-avidity T cells, an activated phenotype, and increased secretion of pro-inflammatory cytokines (reviewed in Legroux and Arbour's [61] study). CD4^+^ T cells, along with CD8^+^ T lymphocytes [151], can release interferon (IFN)-*γ*, causing an autoantigen-specific inflammatory response and myelin destruction [152]. Tumor necrosis factor (TNF), one of the most important inflammatory mediators in a variety of disorders including MS, is increased in the CSF of MS patients. This may be attributed to local glial cells' pro-inflammatory activity or the compartmentalized immune activity of immune cells [153].

The spectrum of T helper (Th)1 and Th2 subsets has now been expanded to include Th17 effector T cells and FoxP3^+^regulatory T cells (Treg) subsets, as well as the lesser studied Tfh, Th9, and Th22 cells [154]. Subpopulations of differentiated CD4^+^ T cells show a high degree of plasticity [155] and although some subsets seem to be fairly stable, some flexibility permits transitions between them and the creation of hybrid transition forms [156]. They can differentiate in response to infection, but can also modify their roles in response to cytokine signaling and other factors [157], and this may result in the preservation of subpopulations with detrimental effects, such as promoting immune pathology.

During the early stages of MS, the activation of myelin-specific T cells is accompanied by the instability of Th1, Th2, and Th17 cytokines [152, 158]. Th1 cells that produce IFN-*γ* and Th17 cells that produce interleukin (IL)-17 are both detrimental, although most investigations have found that Th17 cells are the major perpetrators [146, 147]. CD4^+^ T cells with the pro-inflammatory phenotype marked by Th17 cytokines [159] can proliferate in vitro [160], grow peripherally, avoid tolerance induction, induce neuronal injury indirectly, and become essential players in the development of disease [7]. The pathogenicity of Th17 cells in autoimmune diseases appears to be related to the Th17–Th1 plasticity, which is controlled by the cytokine environment [161], and IL-23/Th17 seems to play a key role in disease progression. In contrast, Th2 cells and Tregs produce anti-inflammatory cytokines and prevent Th1/Th17-mediated immunopathology, Furthermore, Th2 immune responses may promote the development of autoantibodies, which would aid in the pathophysiology of SPMS [154].

CD8^+^ T cells are widely known for their role in viral defence and tumor immunology, but their contribution to autoimmune disease is less well understood [162]. Autoreactive CD8^+^ T cells directed against CNS autoantigens along with CD4^+^ T cells potentially play a part in the development of MS [163, 164]. CD8^+^ T-cells are involved in numerous disease-driving pathways, including pro-inflammatory cytokine production and cytotoxicity. Additionally observed are the production of IFN*γ* and oligoclonal growth, which are common triggering patterns in disease lesions [165]. Cytotoxic CD8^+^ T cells may target oligodendrocytes and perhaps neurons directly, causing demyelination and axonal damage [7]; however, their potential role in MS has only recently started to be recognized where activated, cytokine-producing lymphocytes effectively modulated CNS inflammation. The human leukocyte antigen, or MHC, and its by-products are crucial in determining how the immune system responds. For interactions with CD4^+^ (helper) T cells, the MHC class II region needs to be shared, and for interactions with CD8^+^ (cytotoxic) T cells, the MHC class I region needs to be shared [166]. MHCI molecules are found on all nucleated cells, including those in the CNS, and CD8^+^ T lymphocytes detect antigens presented on them. Persistent antigen stimulation (such as in autoimmunity) results in a restricted expansion of CD8^+^ T cell proliferation rather than cytotoxicity.

Although CD4^+^IFN-*γ* mRNA-expressing lymphocytes are abundant in MS pathology and the HLA class II haplotype is associated with disease [167], increased numbers of CD8^+^IFN-*γ* mRNA-expressing lymphocytes have also been found in MS patients, indicating the involvement of CD8^+^ T lymphocytes in pathology [164]. This is corroborated by the growing genetic data of HLA class I influences [167]. By delivering an MBP self-peptide to T cells, HLA-DR2 can promote both induced and spontaneous neurological illness that resembles MS. However, some genes within the MHCII region on the DR2 haplotype may also contribute to the MS risk [168]. As such, the first modest attack may be initiated by MHCI-restricted CD8^+^ T cells, but disease development likely requires additional contributions from MHCII-restricted CD4^+^ T cells. Some of these recruited lymphocytes may react to the well-known immunodominant CD4^+^ T cell MOG_35–55_ epitope, which is created by the initial inflammation caused by cytotoxic CD8^+^ T cells. Such an epitope is detrimental in MS; as it not only binds the HLA-DRB1 ^*∗*^1501 encoded HLA-DR2 molecule but also activates T cells from MS patients and causes illness in HLADRB1 ^*∗*^1501–transgenic mice [169]. In addition, clonally developed CD8^+^ T cells, plasmablasts, and to a limited extent CD4^+^ T cells, have been detected in CSF of MS patients using single-cell RNA sequencing [63].

Features of activated tissue-resident memory (TRM) T cells were observed in clonally expanded T cells. The TRM-like phenotype was apparent and more pronounced in cells from individuals with confirmed MS, indicating that clonally amplified TRM-like CD8^+^ cells play a significant role in disease pathogenesis [33, 167]. Recent evidence also showed that myelin-specific and nonspecific CD8^+^ T lymphocytes maintain the autoimmune immunopathology that occurs in MS. When animals were given obscure apoptosis-associated epitopes (AEs) and EAE was induced, AE-specific CD8^+^ T lymphocytes with an effector/memory phenotype aggregated in the CNS, exacerbating the disease [170].

## 7. Treg Cells

Although autoimmunity is still considered the key pathological feature of MS, recent research has suggested that autoimmunity mediated by Th2 cells and Tregs may exhibit a protective effect on CNS-damaged tissues [171]. A phase-2 trial with a monoclonal anti-CD4 antibody failed to diminish MS pathology [172], possibly due to CD4^+^Foxp3^+^ Tregs playing a crucial function in inhibiting autoreactive T-cell activity [97]. CD4^+^ Tregs from MS patients have altered functions [173], with myelin-specific T cells having a lower capacity to generate IL-10 than those from healthy controls [174], which is associated with their suppressive role in pathogenicity [159]. Additionally, Bjerg et al. [175] found that a change in Treg frequency is linked to the disability status of patients with RRMS.

During exacerbation of MS, antagonistic CD94/NKG2 receptors may be activated on CD8^+^ T cells, lowering both cytolytic action and suppressive/regulatory potential. Under normal circumstances, CD8^+^ Tregs suppress autoreactive CD4^+^ T cells and when CD8^+^CD25^+^Foxp3^+^ Tregs are added to cultures, they inhibit CD4^+^ self-reactive T cell expansion as well as IFN-*γ* and IL-17 release [176]. As self-reactive T lymphocytes may avoid clonal ablation in the thymus, peripheral tolerance, carried out by Tregs, is critical. Several CD8^+^ Treg subclasses have been identified, and a comparison of MS patients and healthy individuals' blood indicates differences in Treg populations [177] where both immature and active subtypes from those with MS displayed suppression impairments following CD2 stimulation, and that natural Tregs (nTregs) from people affected with MS, were less suppressive than induced Tregs (iTregs) [178]. The activation of an autoimmune response may be aided by a disruption in this regulating mechanism [179]. Recent research highlights the relevance of Tregs in dampening Th1 and Th17 effector cell functions [180, 181] and a correlation between Th17 cells and negative outcomes in MS has been identified. Tregs and Th17 cells play opposing roles, and the balance between them influences the severity of MS. The ratio of Treg to Th17 cells in the peripheral circulation in relapsed MS patients was found to be decreased. Treg percentage and proportion of Treg/Th17 have been inversely linked with clinical characteristics [182]. Treg depletion, malfunction, and instability have all been linked to the development of autoimmune disorders [183] and may act as the driving force behind MS, resulting in immunological disturbances, inflammation, and neuronal injury [180, 184]. This is the case in the development of RRMS [185]. Several miRNAs, including miR-15a, miR-19a, miR-22, miR-210, and miR-223, which negatively regulate gene expression at the mRNA level of the TGF-signaling pathway, important for growth and development of Tregs, were elevated in MS patients' plasma, peripheral blood mononuclear cells, and brain white matter tissues, implying an important role for Tregs in MS pathophysiology [186].

Breaches in both central and peripheral tolerance have been reported in MS. Negative selection is mainly mediated by the autoimmune regulator (AIRE) that regulates intracellular expression of tissue-specific antigens (TSAs) and it also regulates expression of TSAs, distinct from those in the thymus, in the periphery. Transplantation of AIRE-encoding BM cells in mice has been shown to attenuate MOG-induced EAE. Thymocytes receiving TSA-expression levels just below the threshold for negative selection can, with the help of mTECs and myeloid dendritic cells (mDC) cells, become CD4^+^CD25^+^Foxp3^+^ regulatory T cells (nTreg). Additional thymic dysfunction in the form of a defect in global naïve T cell thymic output (identified by TCR excision circles-TREC marker) has been demonstrated in young RRMS patients, who had lower numbers than those in age-matched HC [187], suggesting a premature thymic involution in MS [188]. In addition, the significant decrease in TRECs observed in normal healthy individuals as they age is not seen in individuals with MS. Thymic dysfunction in MS has been recorded using a marker for recent, immature CD4 thymic emigrant (RTEs) naïve T cells, CD31^high^, with a more pronounced defect seen in those with PPMS than RRMS [189]. As a result of the shortage of naive subgroups, MS Tregs are often dysfunctional and overwhelmingly abundant in memory cells that have decreased their innate suppressive capability [190]. The functional inefficiency of Tregs in MS patients is associated with an uneven distribution of naive and memory Treg subsets and many findings have indicated that this aberration is due to the early reduction in thymic-dependent Treg neogenesis. Treg differentiation in the thymus is critically determined by mDCs, and the thymic stromal lymphopoietin receptor (TSLPR) encoded on mDCs is a crucial element of the molecular mechanisms involved in this activity. TSLPR expression on mDCs is reduced in MS patients [191].

Peripheral tolerance is regulated by the interaction of complex T cell-intrinsic mechanisms, involving costimulators, and transcriptional and epigenetic mechanisms, and extrinsic mechanisms, involving Tregs. Alteration of expression of several costimulatory signaling molecules required for T-cell activation, including CD28, ICOS, CTLA4, CD40, PD1, CD137, and CD58, have been associated with MS. CD137, suggested to be involved in impaired regulatory function of Treg and plasmacytoid DC in MS patients, is elevated in MS individuals, while FoxP3 is impaired in RRMS [180]. Observations concerning the frequency of nTregs in the periphery between MS patients and HC are contradictory. No differences [164, 167–169] as well as an increase in their numbers [170, 171] have been reported. In contrast, the numbers of Tregs are consistently found to be increased in the CSF of MS patients compared with HC [169, 172]. Of note, Tregs are scarce in MS brain lesions [97] whereas in EAE, they proliferate into the CNS [173]. CD4^+^ Tregs expressing the transcription factor Foxp3 is required for peripheral immunological tolerance (regulation of autoreactive immune cells) [192]; deletion of Foxp3 on CD4^+^ regulatory cells leads to multiorgan autoimmunity [162] and malfunction of Tregs are related to the alteration of the *Foxp3* gene.

CD4^+^Foxp3^+^ Tregs play an important role in inflammatory disease defence but can also generate pro-inflammatory cytokines. In the case of MS, the data supporting these two alternatives are controversial. Foxp3^+^ Tregs in the brains of MS patients largely release IL-10 and have upregulation of the IL-33 receptor ST2 (linked with significant Treg action), implying that the repressive role of Tregs is maintained in the inflamed brain [98]. Other investigations indicated that Tregs were dramatically reduced in stable MS patients, as characterized by “CD4^+^CD25^+^FOXP3^+”^ [193] and “CD25, CD39, Foxp3, CTLA4, and GITR expression,” but were remarkably recovered to reasonable ranges upon severe clinical onset. So, Tregs do not cause clinical recurrence; instead, they respond to inflammation, to regain stability [99]. Recent research has also revealed that the immunosuppressive function of Tregs is defective in MS patients and the *FOXP3* gene plays an important role in the control of CD4^+^CD25^+^FOXP3^+^ Treg cells. Genetic variations in the *FOXP3* gene's promoter region may affect gene presentation and so influence the disease severity [193–195].

With clinical relapse, MS sufferers have a scarcity of CNS-specific CD8^+^ Tregs. CD8^+^ Tregs derived from the CNS are cytolytic and able to kill harmful CD4^+^ T cells. Such CD8^+^ Tregs are mostly found in the terminally differentiated (CD27^−^, CD45RO^−^) subpopulation with their repression mediated by IFN-*γ*, perforin, and granzyme B (GzmB). A considerable depletion of these terminally specialized CD8^+^ Tregs cells was observed in MS cases of acute recurrence, together with a reduction of perforin and GzmB. The induction of GzmB, by pretreating exacerbation-derived CD8^+^ Tregs with IL-12, was shown to result in a dramatic regain of Treg suppressive function [196]. In another study, CD4^+^ T cells from MS individuals were shown to be resistant to repression by patient-derived or normal donor-derived ex-vivo Tregs. While GzmB has been shown to promote Treg resistance through a novel, apoptosis-independent pathway, memory GzmB was not expressed by CD4^+^CD127^lo^FOXP3^+^ Treg subtypes but was represented by stimulated, nonregulatory CD4^+^ T cells separated from patients with MS [197].

The therapeutic effects of Tregs in MS have been shown following glatiramer acetate (GA, Copaxone) administration to EAE mice, which resulted in increased Tregs, but fewer Th17 cells, and a reduction in CNS lesions, even if treatment followed exacerbation of disease [198]. Treg cells, stimulated by antiCD40 Ab or 8-oxo-dG, inhibited the expression of CCL2/CCR2, VCAM-1, PECAM-1, and Act1 in mast cells (MCs), which are reactivated by cytokines, particularly IL-17, suggesting their potential to slow EAE progression [199]. Th17 and Treg cells adapt to various physiological conditions during immune response, potentially playing a significant role in autoimmune disease. It has recently been shown that in the EAE model, CXCR3 ligands enhance the polarization of naïve and effector T cells. Effector Th1 pro-inflammatory cell polarization was enhanced by CXCL10/CXCR3 interactions [200] via STAT1, STAT4, and STAT5 phosphorylation while the polarization of CD4^+^ T cells was skewed into Treg-like cells by CXCL11/CXCR3 interaction, mediated via p70 ldnase/mTOR in STAT3- and STAT6-dependent pathways. The resultant Tregs suppressed EAE in an IL-10-dependent manner. CXCL11-Ig fusion protein administered during the initial episode of relapse resulted in rapid remission and prevented further relapse in SJL/J mice while C57BL/6 mice deficient in functional CXCL11, showed rapid suppression of signs of EAE when very low doses of CXCL11 were administered [200].

## 8. B Cells

In addition to producing antibodies, B cells also play roles in the induction, maintenance, and reactivation of CD4^+^ T cells; they present antigens that are necessary for memory cell maintenance and reactivation and influence the function of Tregs [201, 202]. It has been demonstrated that reducing B cell numbers increases the number of Tregs [203, 204]. This could be a contributing factor to the decrease in activated T cells and B cells in the CSF that occurs after anti-CD20 mAb therapy [67, 68]. In the pathophysiology of MS, B cells may play a significant role [14] utilizing both antigen presentation and immunoregulation [205]. Using adoptive transfer models, EAE was shown to be inducible in mice whose B cells were the sole MHCII-expressing APC, and that inducing MHCII on B cells, following the transfer of encephalitogenic CD4^+^ T cells, caused rapid and robust disease onset when compared to mice where MHCII induction was on a normal complement of APCs [206]. B cells are normally found in only small numbers in healthy brains, but their numbers are dramatically increased during inflammation [69]. They are present in inflamed MS CNS tissue [70] and in increased numbers in the CSF of MS individuals. B cell trafficking is influenced by many chemokines, including CXCL13, which is elevated in MS patients, correlating with the increased B cell numbers, as well as with conversion from clinically isolated syndrome (CIS) to definitive MS [207]. As proliferating B cells are seen in intrameningeal follicles of MS patients and there is a predominance of B cell aggregations that are germinal-center-like (larger and more metabolically active), it is likely a site for B cell selection and generation of high-affinity antibodies [71]. Blocking CXCL13 disrupts GC-like structures in non-obese diabetic mouse pancreatic islets, suggesting that these aggregates in MS patients may also arise from migrating memory B cells. Intrathecal immunoglobulin G (IgG) synthesis, CSF oligoclonal IgG bands (OCBs), and lesional IgG deposition are pivotal features of disease in MS. Plasma IgG antibodies form large aggregates (>100 nm), inducing complement-dependent apoptosis in MS neuronal cells [72]. The development of ectopic lymphoid follicles in the meninges of MS patients may therefore be a crucial step in fostering humoral autoimmunity and in the worsening of the condition [71].

Even though OCBs in the cerebral fluid have been a diagnostic marker for MS for over 3 decades, the role of antibody-secreting cells in the disease remains unknown. There is growing evidence that plasma cells play a role in the release of autoantibodies. Nonproliferating plasma cells (CD138^+^Ki67^−^) were found in the parenchyma of brain samples from patients with MS. In EAE, plasma cells were discovered in the inflamed spinal cord's meninges and parenchyma, surrounded by tissue patches that resembled survival habitats for these cells, with upregulation of chemokines (CXCL12), adhesion molecules (VCAM1), and survival factors (APRIL and BAFF) [73]. Another study showed that brain gray matter (GM) antigens are detected in preclinical MS patients by augmented VH4^+^ peripheral plasmablasts that overuse immunoglobulin heavy chain V-region subgroup 4 (VH4) genes and produce antibodies that recognize intracellular antigens of neurons and astrocytes [74]. Peripheral plasmablasts may therefore play a role in the autoimmune response linked with MS and offer a way to look at the advancement of autoreactive B lymphocytes at the time of the first clinical episode.

B lymphocytes and plasma cells are found at active lesions in both MS and EAE and antibodies have been located where demyelination has occurred [75, 76]. Clinical trials using CD20-targeted monoclonal antibodies (mAbs) in patients with RRMS and PPMS confirm B cells' critical role in MS pathogenesis, with plasma B cells producing antibodies identified as OCBs in the CSF. Although CSF OCBs are not specific for MS, they point to a source of IgG in the CNS of MS patients [208]. The effectiveness of B cell reduction therapy and the discovery of leptomeningeal ectopic lymphoid tissue (ELT) in MS patients rekindled interest in B cells' antibody-independent harmful activities in neuroinflammation [209].

Chronic Epstein–Barr virus (EBV) infection also targets B cells. When B cells are infected with EBV, their differentiation is disrupted, and they become immortal. The presence of EBV-infected B cells causes demyelination and axonal loss in the cortex [77]. A comparison of C57BL/6 (B6) mice actively immunized with MOG_35–55_, to induce T cell-dependent EAE, with mice immunized with MBP-proteolipid protein fusion protein (MP4), to induce additional B cell dependence, indicated the presence of CD3^+^ T cells with few/no B220^+^ B cells in diffuse infiltrates, while B cell aggregates had significant B cell infiltration and only a few T cells [78, 210]. Neurodegeneration is linked to high levels of meningeal inflammatory activity in GM pathology of MS, and ectopic lymphoid follicles (eLFs) and B cell aggregates have been detected in the inflamed meninges of some patients with PPMS [78]. B cells play an important role in MS pathology, and clinically relevant pathways show a link between B cell inflammation in the forebrain and the spinal cord, as well as between B cell inflammation of the spinal meninges and lymphocyte infiltrates, demyelination, and axon loss. It suggests that B lymphocytes play a pivotal role in maintaining inflammation and tissue damage throughout the CNS as the disease progresses [70]. In pertussis toxin (PTX)-induced Th17-EAE, B cell insufficiency reduces paralysis in mice, indicating that B cells are important for inducing demyelination. PTX also promotes an inflammatory B cell phenotype in the periphery, allowing pathogenic B cells to accumulate in the CNS, where they are associated with the duration of disease in animals with milder EAE [79].

At the early stages of clinical illness in MS, CD19^+^ B cells tend to have more widespread hypomethylation than T cells or monocytes. This epigenetic pattern is associated with B cell differentiation and overactivation, suggesting that abnormal B cell function may play a role in MS pathogenesis [211], resulting in memory B cells that are pathogenic in MS, invading the brain and evolving into antibody-secreting cells [80]. It has been proposed that the subpopulations of naïve and memory B-cells produce different effector cytokines; IL-10 is almost exclusively produced by Bnaïve B cells, while pro-inflammatory cytokines, such as TNF-*α* and lymphotoxin, are mainly produced by Bmem B cells [203, 204]. The percentage of CD86^+^ Bnaïve cells was found to be significantly higher in untreated RRMS patients than in those receiving IFN treatment or in control subjects [212]. Depending on the exact expression patterns of receptors and ligands as well as the levels of activation, CD86 on B cells can bind to either CD28 or CTLA-4 on T cells, with resulting costimulation or coinhibition [213].

B cells are becoming more widely recognized as highly efficient APCs, capturing antigens through their membrane-bound B cell receptor, processing them, and displaying them on their cell surface cross-linked with MHCII molecules. Moreover, B cells are a major source of cytokines which influence the strength and quality of Th cell responses [162]. IL-6 produced from B cells provoked pathogenesis in EAE, whereas IL-10-producing B cells protected against autoimmunity in EAE. Mice with IL-10-deficient B cells also exhibited normal activation of CD4^+^Foxp3^+^ Treg cells and acquired effective protection from illness following adoptive transfer of B cells extracted from wild-type (WT) mice post-EAE recovery. This produces evidence of an independent pathway of protection against CNS autoimmunity, as well as therapeutic prospects in the treatment of MS [214]. Many treatment approaches are being studied to help improve prognosis, prevent relapse, and lessen the level of disability. Most MS treatments alter B cell trafficking, phenotype, or frequency in one way or another [215]. The pro-inflammatory responses of myeloid cells and CD4^+^ and CD8^+^ T cells are significantly reduced when B-cells are reduced [216, 217]. Anti-CD20 monoclonal antibodies, including rituximab, ocrelizumab, ublituximab, and ofatumumab, have been developed for the treatment of RMS [215]. Since plasma cells do not express CD20, memory B cells are believed to be the primary target of these anti-CD20 antibodies in MS [218]. Rituximab and ocrelizumab are effective targets for PPMS treatment [219, 220], while small-molecule drugs like evobrutinib may enhance BBB penetration and treatment initiation and termination [221].

## 9. Macrophages and Microglia

MS pathophysiology requires both innate and adaptive immune cell activation [222]. Although many cell types in the nervous system engage in innate immune reactions, it is the macrophages and microglia that are the most notable innate mediators of pathological alterations in MS [162]. Macrophages are phagocytic cells that include tissue-resident groups as well as circulating monocyte-derived subsets. Microglia are CNS resident phagocytic cells that play a vital role in maintaining CNS integrity in a steady state by removing damaged or unneeded neurons and synapses. In the presence of inflammatory or pathogenic stimuli, microglia and CNS-infiltrating macrophages not only govern innate immunity components but also modulate adaptive immune responses, and many CNS illnesses are caused by dysregulation of these reactions [81]. Indeed, dysregulation of peripheral pro-inflammatory monocytes/macrophages has the potential to initiate organ-specific autoimmunity by penetrating target organs nonspecifically, thereby causing the expansion of preexisting autoantigen T cell populations, in genetically predisposed individuals [223]. Pro-inflammatory cytokines and the reactive oxygen species, NO, and other oxygen radicals produced from active microglia, impair axonal and synaptic function, and stability and prolong the neurodegeneration [7]. As mitochondrial damage may be a key cause of oxidative stress, it may also play a role in the generation of inflammatory processes in MS [224]. By directing and activating T cells as well as producing a pro-inflammatory environment in the CNS, activated microglia and macrophages likely trigger neurotoxicity [7], while local microglia, being efficient APCs, conceivably play a crucial role in antigen presentation at the onset of clinical illness and contribute to the breakdown of functional myelin in MS [82]. A model of early retinal neurodegeneration, one of the first clinical indications of MS, indicated that the retina had the most rapid initiation of local microglia, and the optic nerve had the largest proportion of activated microglia [92].

A recent study indicated that endogenous immunological cycles may produce seasonal changes in EAE intensity and, potentially in the development of MS, that may be associated with macrophage/microglia functions [225]. Differences have been observed between active, mixed active/inactive, and inactive lesions based on macrophage/microglial (inflammatory activity) distribution and demyelinating activity. While macrophages/microglia were found traversing active lesions, in mixed active/inactive lesions macrophages/microglia were restricted to the lesion perimeter, while inactive lesions are virtually entirely devoid of macrophages/microglia [83]. Whether microglial activation is detrimental or beneficial in MS has long been questioned. On the one hand, they play an important role in inflammation and demyelination. Here, cytokines and chemokine-like neuroinflammatory mediators are released leading to the destruction and engulfment of the myelin sheath, through the presentation of myelin-derived antigen to autoreactive T cells [101, 226, 227] and the induced maturation and activation of encephalitogenic Th17 cells, particularly by exposing them to IL-6, IL-23, IL1, and TGF [228]. On the other hand, they can be modulated to produce neuroprotective mediators, for example, *α* B-crystallin (HSPB5), an endogenous agonist for TLR2 in CD14^+^ cells, activates a neuroprotective glial cell response [226].

Microglia-depleted mice have delayed EAE onset, and a reduction in disease severity and inflammation, providing further evidence of their critical role in MS pathogenesis [81]. While macrophage infiltration is a characteristic feature of MS, the disruption of M1/M2 balance is involved in disease progression. In MOG_35–55_-induced EAE, there was a shift to increased pro-inflammatory M1 phenotype as evidenced by expression of activation/costimulatory markers, iNOS, CD40, CD80, IL6, IL12, CCL2, and CXCL10, while the M2 subset was depleted (CD206 and CCL22 were downregulated in favor of several M1 marker upregulations) [93]. Oligodendrocyte loss is a common characteristic in the tissue surrounding quickly developing MS lesions, and macrophage activity is generally concerned with the removal of defective or decaying myelin [84]. The morphological changes and expansion of cell populations in microglia can be a result of elevated levels of IFN-*γ*, which culminate in the upregulation of activation markers, including MHCI-II, CD86, IL6, and iNOS, that may contribute to BBB leakage and/or T cell infiltration, resulting in cognitive impairment [229]. In addition, proliferative T cells need communication with myelin antigen for reactivation, a function performed by the antigen presentation capability of macrophages and microglia. This nonspecific immune interaction with the acquired immune system at the site of inflammation of the CNS is key in MS initiation [85]. Indeed, the aggregation of macrophages/microglia in the MS brain is regarded as the primary aggressor in the onset of neurodegeneration [86].

Numerous investigations have also found that macrophages/microglia can play beneficial roles by supporting remyelination, eliminating or inhibiting myelin debris, and releasing neurotrophic substances [230]. In MS, upon ingestion of myelin particles, activated macrophages acquire a foamy appearance [231] akin to anti-inflammatory M2 macrophages and are therefore likely to contribute to inflammation reduction, and aid in lesion recovery [232]. In addition, Galectin-4, a unique negative soluble regulator in the timing of embryonic myelination and oligodendrocyte separation, is found in axons and microglia/macrophages in MS lesions. Activated microglia/macrophages, therefore, possess both beneficial and potentially harmful impacts in MS, perhaps explaining some of the disease's relapsing–remitting characteristics [233].

Analysis of whole cryopreserved MS plaques, with monoclonal antibodies for macrophage, monocyte, and MHCII selectivity, revealed that those from acute MS patients had lymphocytic perivascular invasion, plaque hypercellularity, plaque macrophage penetration, and intramacrophage myelin degradation [87]. Macrophages leaving the bloodstream metabolize myelin either by pathological destruction or by initiating signals in the vascular area or the plaque [88]. Interestingly, low-grade active demyelination of an unusual type (frustrated phagocytosis) was identified in two SPMS WM autopsy sections. Here traditional microglial clusters were interacting with damaged myelin, coupled with C3d deposition, but the lengths were too large to be phagocytosed [234]. Macrophages/microglia were found to assemble in periplaque demyelinated lesions in the spinal cord of progressive MS patients, but with limited phagocytic activity targeting myelin fragments, and showing low inflammation causing a gradual degeneration of myelin and the absence of remyelination [94]. In patients with subpial lesions, there was an increase in the frequency of activated (CD68^+^) microglia/macrophages, while the number of neurons was significantly diminished in acute MS, usually occurring in lesion GM. Meningeal inflammation and lymphoid-like formations were observed in acute MS, the degree of which was associated with microglial/macrophage activation but not with the region of cortex demyelination. This was reflective of the presence of lymphoid-like shapes next to GM lesions along with areas of incompletely demyelinated/remyelinated cortex GM [95].

Microglia display a distinct transcriptional profile and surface protein expression pattern (discrete molecular homeostatic “signature”) in the healthy brain, different from tissue macrophages [96]. They exhibit both phenotypic and functional plasticity in healthy and diseased brains. One of the main indicators of neurodegeneration, including in MS, is persistently activated inflammatory microglia. In contrast, the resolution of inflammation and the promotion of remyelination depend on microglial phagocytosis of myelin debris. The aberrant activation and promotion of specific polarization states to modulate activity in neurodegeneration and inflammation may be caused by the dysregulation of lesion-specific miRNAs [235] and circulating biomarkers. The precise function of miRNAs in microglia that aid in the advancement of MS though remains mostly unclear.

Postmortem analysis of MS brain NAWM microglia indicates that they are of an activated phenotype, characterized by enhanced genetic expression associated with inflammation and cellular stress, likely due to continued response to neuroinflammation. However, they exhibit an immunosuppressive trait [236]. Single-cell RNA sequencing has confirmed this stress response in NAWM brain macrophages and detected distinctive microglia and macrophage populations during discrete stages of neurodegeneration [237].

While there has been considerable debate over the years as to whether monocytes are only perivascular, and microglia the invading mononuclear cells in active lesions, recent research confirms the abundant presence of active blood-borne monocytes in MS lesions [90]. Furthermore, levels of IL-6 and IL-12 secreting monocytes were higher in patients with MS than in healthy individuals or those with another neurological disease, and the monocytes displayed increased CD86 expression that correlated with disease duration.

Gene expression changes identified in MS lesions may help elucidate monocyte/microglial functional roles in MS. The excitation of the inducible NF-*κ*B group in macrophages in MS plaques possibly increases the inflammatory processes by overexpressing NF-*κ*B-controlled surface molecules and cytokines [238]. On the other hand, low-density lipoprotein receptor-related protein-1 (LRP1) linked to several processes, including intracellular signaling, lipid metabolism, and apoptotic clearance, as well as having a role in the regulation of the production and degradation of amyloid-*β* and the internalization of ApoE in Alzheimer's disease, is elevated in expression in MS lesions compared to healthy tissue, where it is thought to act as a microglia anti-inflammatory and neuroprotective stabilizer [239]. Golli proteins, which are chemically identical to MBPs, have been found in mature oligodendrocyte precursor cells (OPCs), activated microglia/macrophages, and certain demyelinated axons in the vicinity of MS lesions. Their presence in mature OPCs supports remyelination attempts, whereas their presence in the microglia/macrophage subset demonstrates functions in MS immunological processes [240]. The proteinase-activated receptors (PARs) are widely known for their immunoregulatory role in inflammation and neurodegeneration. PAR2 is increased in astrocytes and infiltrating macrophages in human MS and CNS white matter in EAE mice, and its activation in macrophages has resulted in the release of soluble oligodendrocyte cytotoxins. PAR2 WT mice with EAE had significantly higher microglial activation and T lymphocyte infiltration, as well as increased demyelination and axonal damage in the CNS [241] compared to mice lacking PAR2 expression. Furthermore, autotaxin (*ATX*) genetic ablation from CD11b^+^ cells reduced the burden of EAE, implying that the *ATX*/lysophosphatidic acid (LPA) axis also plays a harmful role in neuroinflammatory diseases. Increased levels of ATX/LPA have been found in the plasma and spinal cords of EAE mice [242]. *ATX* regulation is not fully understood, but TNF and IL-6 have been reported to increase their expression. *ATX* expression from macrophages may be central to EAE pathogenesis through their stimulation and effector functions, while *ATX* expression from microglia is thought to have a more beneficial role, possibly promoting wound healing and recovery from disease [242].

## 10. Dendritic Cells

Dendritic cells (DCs) are found in healthy CNS tissue where they have the potential to sample CNS antigens and direct T cell functions. Several EAE studies indicate that DCs within MS lesions are functionally abnormal. Different classes of DCs have been described [101, 102, 243], CD8a^−^CD205^+^DCs have been shown to display an intrinsic propensity for crosspresentation to CD8^+^ T cells, while CD8a^−^33D1^+^DCs are better at MHCII presentation to CD4^+^ T cells. A population of conventional DCs were identified as licensing T cells to initiate inflammation [102], implicating them as important determinants of CNS autoimmune events. Concerns about the actual function of DCs in autoimmunity remain unresolved, despite implications for DCs contributing to pathophysiology arising during clinical trials and experimental models [244]. According to research in humans and experimental disease models, natural DCs (nDCs) derived from monocytes have recently emerged as essential inducers of the immunopathological chain in MS. The density of DCs in the brain parenchyma is modest under healthy settings but increases during neuropathology. Myelin-reactive T cells require the involvement of CNS microvessel-associated DCs to recognize the local antigen. In both the induction and effector phases of EAE, the active state and compartmental dispersion of DCs generated from the CNS and related lymphatics appear to be the limiting determinants [103], and DCs control and optimize recruitment of encephalitogenic and Tregs into the CNS. DC surface expression of costimulatory or coinhibitory molecules has a significant impact on this ability. In EAE, expression of TLR7 of DCs was found to be increased which ultimately exacerbated the disease by activating MOG-distinct T cells, causing elevated autoantibodies in the bloodstream that intensified inflammation in the CNS and repressed Foxp3^+^ Tregs both in the CNS and the periphery [104].

In MS, DC activation is interrupted by the phagocytosis of myelin which may also alter their capability to trigger the allogeneic T cells activation. TGF-*β*1 can be increased and CCR7 downregulated without hampering the differentiation into Th cell subsets. Thus, myelin-phagocytosing DCs have a profound role in immune regulation in MS [101].

## 11. Neutrophils

The use of preclinical animal disease models, as well as human sample analyses, revealed that neutrophils have a wide range of effector functions that contribute to MS pathogenesis, including secretion of inflammatory mediators and enzymes such as IL-1 [105], elimination and phagocytosis of myelin (as debris), discharge of neutrophil extracellular baits, generation of reactive oxygen species (ROS) [106, 107], breakdown of the BBB, and production and introduction of autoantigens [108]. Numerous cytokines and chemokines promote the migration and proliferation of neutrophils and lymphocytes to the CNS in MS [109]. When PwMS relapse, neutrophils have been found in their cerebral fluid at the early stages of the disease, which suggests a connection between neutrophils and IL-17A levels [110]. Neutrophils, as well as Th17 immune responses, may be more crucial for the induction of MS than for disease development and progression [110]. An increase in neutrophil number and priming, has been observed in MS patients compared to healthy individuals, characterised by reduced apoptosis, augmented degranulation and oxidative burst, and increased number of neutrophil traps, coupled with increased expression of TLR-2, CD43, IL-8R and FPR1 [107, 111], and complement anaphylatoxins, C5a and C3a, as well as C3aR, a receptor extensively expressed by neutrophils during meningitis infection, at sites of inflammation. Since the number of neutrophils in the CSF tends to decrease as the disease progresses, the innate immune system may become active in the early stages of adulthood [110]. Postmortem CNS material from an acutely ill MS patient showed neutrophil infiltration in BBB-leaking regions [112]. Neutrophil extracellular traps (NETs) have also been shown to control adaptive immune cell action [245] by effectively catching and destroying pathogens while activating DCs and priming T lymphocytes. An increased number of circulating NETs was observed, particularly in males, in some RRMS patients and those with other MS subtypes, compared to HCs [106]. However, unlike their documented role in other autoimmune diseases, the investigators found no association with increased neutrophil priming, arguing against a major role in MS pathogenesis, although this may merely be reflective of the methodological approach and further investigation may clarify this.

There is growing evidence for a role for IL-1, produced mainly by neutrophils and monocyte-derived macrophages (MDMs), in MS and EAE. IL-1R1 induction on radiation-resistant cells by IL1*β* was required to initiate disease, while neuroinflammation in EAE was triggered by IL-1*β*-dependent paracrine linkage between infiltrating neutrophils/MDMs and pial venous plexus endothelial cells (ECs) [105]. There have been observations of neutrophil presence prior to B cell cluster development, in the subarachnoid region of the spinal cord in EAE, and CXCR2-mediated granulocyte trafficking to the CNS reduced the number of pathogenic B cell clusters and the severity of disease. EAE dependent on B cell antigen presentation was also abolished by B cell-restricted very late antigen-4 (VLA-4) impairment. Neutrophils coordinate VLA-4-dependent B cell accumulation within the meninges during neuroinflammation, a critical early stage in the development of ELT in MS [209]. The neutrophil-to-lymphocyte ratio (NLR) in peripheral blood has been studied concerning several autoimmune disorders, and in RRMS patients, an increased NLR is linked to disease activity at the time of initiation [246], while NLR and monocyte-to-lymphocyte ratio (MLR) are both linked to neurological impairment and brain atrophy. These ratios may reflect a hematopoietic bias toward improved productivity and pro-inflammatory priming of the myeloid innate immune system in combination with dysregulated adaptive immune processes and thereby serve as a complementary and self-reliant indicator of the severity of MS-related neurological dysfunction and MRI findings [247]. The neutrophil subpopulation increases and the lymphocyte compartment decreases in individuals with inactive RRMS, indicating a potential regulatory role for neutrophils [248]. Neutrophils and their by-products can also actively contribute to the reduction of inflammation through a variety of mechanisms. The first step is to produce lipoxins, resolvins, and protectins [249] to promote neutrophil absorption by macrophages and inhibit neutrophil infiltration. After that, inflammatory chemokines and cytokines are scavenged by scavenger and decoy receptors [250]. Lastly, macrophages efferocytose apoptotic neutrophils, converting them into an M2-like state thereby reducing inflammation [251].

## 12. Natural Killer Cells

Natural killer (NK) cells have recently received a lot of attention for their role in autoimmune regulation [252]. They are both powerful cytotoxic killers and unique immune regulators that function via activating and inhibitory cell surface receptors and thus form a mechanistic link between the innate and adaptive immune system [62, 253]. In individuals with acute phase MS, NK cells in peripheral blood were found to be increased compared to that of HCs [113]; however, NK cells' inflammatory and autoimmune reactions in the CNS differed significantly from those in the periphery. The accumulation of NK cells, in a mouse model of disease, resulted in improved disease outcome, but selective inhibition of NK cell trafficking to the CNS worsened symptoms, indicating reliance on CNS-resident rather than peripheral NK cell activity for disease. Interactions with microglia and inhibition of myelin-reactive Th17 cells form part of CNS-resident NK cells' function [254]. Even in the absence of the traditional complement system, conformation-specific antimyelin antibodies contributed to cortical demyelination. However, when there was a pathogenic antibody response, NK cells were important for perivascular cortical demyelination, consistent with their predominate perivascular location in demyelinated cortical lesions of MS patients [255].

NK cells can facilitate host resistance without being presensitized by antigens and can cause severe destruction of cells with abnormal expression of MHC I molecules [256]. There is evidence to indicate that NK cells can contribute to MS development [257], CD16^bright^ CD56^dim^ NK cells appear to act as effective cytolytic effectors, while the immunoregulatory role of CD16^dim^ CD56^bright^ NK cells indicates that they may also act to protect neurons [258]. Given the heterogeneity of NK cells, it is still unclear as to whether the dual roles are due to separate NK cell subgroup functions or based on tissue localization [259, 260]. IFN-*β* can expand regulatory CD56^bright^ NK cells in MS, and in vitro tests show that IFN-*β* and IFN-*β* plus corticosteroids increased the number of Ki-67^+^ NK cells in MS treatment [261]. Plantone et al. [114] found that patients with PPMS and SPMS have a greater percentage of circulating CD3^−^CD56^dim^perforin^+^ NK cells than healthy individuals, implying that this subpopulation may play a role in the underlying aetiology of disease. Another finding indicated that NK cell subtypes do not develop uniformly in all inflammatory neurological diseases, and regulatory CD56^bright^ NK cells and natural killer T (NKT) cells may emerge in the CSF of MS patients as a response to the disease's CNS immune response [115]. The frequency of NK cells in the CSF of MS patients was lower than in blood. During neuroinflammation, CSF NK cells have an immature phenotype with strong expression of CD56 and CD27, and low CX3CR1 expression, in contrast to blood NK cells. This indicates that the CSF may act as a gateway for NK cell migration and maturation before infiltration of the CNS [116].

NK cells show various immunoregulatory functions through the elimination of immature myeloid DCs, and the selection of those best for T cell priming. They can also boost plasmacytoid DC IFN-*α* production and can be activated by mature DCs to release IFN-*γ*, which modulates Th1 priming, CD8^+^ T cell response, and the DC-induced polarization of naive T cells. They can destroy virally infected, stressed, or neoplastic cells, as well as mediate the death of activated T cells, APCs, and endothelial cells. They can also generate immunological memory responses that may be antigen-specific or nonspecific and provide increased pathogen protection [134].

In CNS autoimmunity, NK cells play an important role in regulating T cell activity. In MS, the DNAX accessory molecule-1/CD155 bond of NK cells and CD4^+^ T cells is disrupted, resulting in instability of NK-mediated T cell activity [252]. Pathogenesis may be linked to the Th17 cytokine released by MS KIR2DL2^+^NK cells in the presence of HSV-1 infection [262]. TGF-*β*, important for Th17/Treg proliferation, has been linked to the activation of NK cells in EAE and may protect against disease in mice by increasing proliferation of NK cells [263]. MS patients treated with IFN-*β* or natalizumab showed increased NK cell receptor (NCR1) intensities [264].

Through an NKG2D signaling pathway, NK cells contribute to the rejection of allogeneic neural progenitor cells (NPCs) in a viral-induced demyelination paradigm [265]. In MS, NK cells are thought to have an immunomodulatory influence, and the NKG2C^+^ subset amplification has recently been linked to delayed regeneration [266]. Patients in remission and those with CIS had significantly higher migration rates of NK cells (CD45^+^CD3^−^CD16/56^+^ and CD3^−^CD16/56^+^CXCR4^+^) than patients in relapse and healthy controls [267]. In both humans and animals, NK cells persist in the brain's subventricular zone (SVZ) during the chronic phase of MS, close to SVZ neural stem cells (NSCs), which synthesize IL-15 and maintain viable NK cells. Liu et al. [117] observed that NK cells reduced NSC repair following neuroinflammation, implying that NSCs and NK cells have mutual connections that govern neurorepair.

## 13. Mast Cells

MCs are found in the brain's thalamus and hypothalamus and produce histamine, which exist before any pathological or clinical symptoms of MS [119]. These cells play a critical role in allergic [268] and anaphylactic events involving immunoglobulin E (IgE) [269] and have increasingly been linked to inflammatory disorders in which they are induced by non-allergic stimuli including neuropeptides and cytokines, frequently with synergistic effects like IL-33. MCs are capable of preferentially releasing pro-inflammatory molecules without degranulation and many inflammatory conditions, including MS, engage them to bridge with T cells [270]. Their contribution to MS remains controversial [271] with some studies indicating a deleterious and pro-inflammatory role, while others challenge their significance in the pathogenesis of MS and EAE [269]. There is evidence that MCs can cause disease in a PTX-free EAE model involving the *c-Kit* gene. SJL-KitW/W-v mice have a deficit of MCs, and present with anaemia and neutropenia but have typical T cell compartments, and a relapsing–remitting course of disease, that can be reverted by restoration of specific MCs [272]. MCs have been seen in the chiasma region and adjacent areas of the optic nerves and tracts in MS [120], where they were dispersed in the parenchyma particularly in and around chronic, active plaques. The detection of IgE on and within MCs indicates that MCs' role in MS pathogenesis may be mediated by IgE. Histological studies indicated that there was an increased number of MCs in the brains of MS patients. The elevated levels of two prominent MC chemotactic factors, CCL5 and stem cell factor, were significantly associated with the levels of MC-specific transcripts, but the stage of inflammatory MS lesions had little impact on the deposition of MCs [273]. Most MCs were grouped in key locations of oedema development in the brain, implying a role in their etiology and subsequent myelin degeneration in MS [274]. MCs appear necessary for both the inductive and effector phases of EAE. Research indicates their requirement for optimum stimulation of autoreactive T cells while their repopulation in the periphery, but not the CNS, results in a more severe disease course [268].

Neuronal elements, such as substance P, MBP, and corticotropin-releasing hormone, generated by stressors, stimulate brain MCs, which can take part in the degeneration of neuronal cells and the myelin sheath [275] by producing various pro-inflammatory molecules and vasoactive chemicals that can break the BBB and excite T cells encountered [270, 276]. Angiogenic factors housed in MC granules may play a significant role in the vasoproliferative interactions taking place in such pathological situations [277]. MCs in the meninges play a vital role in the deposition of antigen-specific Th cells and the expression of GM-CSF. The cells do not concentrate in the meninges nor release GM-CSF in the absence of MCs. Colocalization of MCs and T cells in the meninges and the CNS of newly diagnosed severe MS patients suggests that similar interactions may occur in human demyelinating autoimmune disease [121]. Human MCs release matrix metalloproteinase-9 and IL-6 in response to activated T cells, and myelin, which is partially regulated by TNF *α*, stimulates MCs in a way that increases the number of activated T cells. MS [278], a Th1-cell-mediated illness, displays an inflammatory response that includes the production of cytokines and MC mediators in addition to other inflammatory cells like lymphocytes and macrophages. Though it is widely acknowledged that MC-mediated demyelination, which is brought on by an auto-antigen specific to myelin, requires T-cells [279].

## 14. Astrocytes

Astrocytes, the most abundant cells in the CNS, play significant roles in demyelinating diseases and are largely considered active participants in the MS disease process [280], demonstrating diverse roles in lesion development during the course of MS [281]. While not considered a component of the innate nor adaptive immune system, they exhibit similar activity to immune cells, as they utilize the secretion of various pro-inflammatory and chemotactic cytokines to exert a function in neurodegeneration [282]. In MS and EAE, underlying mechanisms, such as the acquisition of pro-inflammatory monocytes, the formation of neurotoxic and inflammatory elements, and the reduced levels of neurotrophic factors and neuron-support metabolites, promote astrocyte activation states that augment CNS pathology. Conversely, NK cell and Treg-derived cytokines increase astrocyte anti-inflammatory processes [283]. Antibody-mediated pathology also appears to have a role in MS, as indicated by intrathecal IgG production, CSF OCBs, and lesional IgG accumulation [284]. When myelin-specific and certain astrocyte/neuronal-specific MS rAbs were added to spinal cord explant cultures in the presence of complement, they caused considerable myelin degradation and astrocyte stimulation [122].

Astrocytes are heterogeneous, with at least five different populations described, and they appear in different frequencies, volumes, orientations and arborization, dependant on location, and exhibit a broad range of functions including ion and water homeostasis, neurotransmitter recycling, BBB formation/maintenance, immune signaling, and regulation of neuronal synaptogenesis [123]. While astrocytes nourish BBB endothelium and maintain its integrity by releasing tropic factors interacting with endothelial cells end-feet around small vessels [285], the sphingosine 1-phosphate (S1P) molecule, considered to regulate BBB normal structure, is overexpressed on astrocytes around MS lesions, and modulation of S1P receptor signaling on astrocytes appears to dramatically alter the course of disease [286]. The contribution of reactive astrocytes to BBB breakdown during neuroinflammation was demonstrated in angiotensinogen (AGT)-defective rats where significant suppression of AGT was pro-inflammatory cytokine-dependent and associated with occludin under-expression on BBB-ECs [285].

The contribution of reactive astrocytes to BBB breakdown during neuroinflammation was demonstrated in angiotensinogen (AGT)-defective rats where significant suppression of AGT was pro-inflammatory cytokine-dependent and associated with occludin under-expression on BBB-ECs [287]. However, demyelination can be triggered by astrocyte depletion [288] and malfunction [289]. Systemic high mobility group box 1 (HMGB1) is a ubiquitous nuclear protein released by glia and neurons upon inflammasome activation and it activates receptors for advanced glycation end products (RAGE) and TLR 4 on the target cells. The increased expression on astrocytes resulted in disease progression while blocking it in the CNS reduced EAE intensity [290]. Chromogranin A (CgA) and clusterin (CLU), thought to be disease-related, neuro-inflammatory particles and possible CSF indicators, were upregulated in activated astrocytes in MSWM lesions [124]. Immune and/or CNS signaling caused changes in astrocyte-neuron lactate shuttle (ANLS) and glutamate-glutamine cycle (GGC) gene regulation in the MS NAGM, possibly explaining cortical brain abnormality resulting in clinical symptoms such as convulsions, tiredness, and cognitive failure [125].

EAE pathogenesis is characterized by a colossal aggregation of reactive astrocytes [291] associated with upregulated glial fibrillary acidic protein (GFAP) [292], and vimentin [293]. Astrocyte loss resulted in the worsening of symptoms in a stage-dependent manner in Theiler`s murine encephalomyelitis, together with dysregulation of Aquaporin-4 (AQP4). AQP4 is extensively depleted in GFAB-positive hypertrophic astrocytes, not only in demyelinated but also in myelinated layers of simultaneously degenerative neurological lesions [294] and it's linked to structural changes and the appearance of perivascular astrocytes [281]. Altered responses to miRNA involved with the regulation of AQP4 (-100, -145, -320) and glutamate transport/apoptosis/neuroprotection (-124a, -181a, and -29a) have been associated with MS [280].

During demyelination, astrocytes generate significant quantities of matrix metalloproteinases (MMPs), that aid tissue damage and reorganization, produce gliotic scars in inactive lesions, and help maintain the BBB and the blood-spinal cord barrier, as well as remyelination and neuronal integrity [126]. While new remyelinating oligodendrocytes and Schwann cells are primarily formed from adult oligodendrocyte precursor cells, new astrocytes are primarily derived from other preexisting FGFR3-expressing adult white matter astrocytes during the restoration of demyelinating lesions in MS [127]. In MS pathogenesis, a reciprocal impact of astrocytes and lymphocytes in the BBB may boost MMP-2 expression while decreasing IL-17 and IFN-*γ* secretion, critical in sustaining BBB functionality and structure [295]. In addition, the connexin gap junction building block protein, Cx43, with important roles in maintaining myelin sheath and neuronal function, is extensively decreased in acute demyelinating lesion astrocytes [296].

Activated astrocytes expressing CCL20 result in a pro-inflammatory effect in EAE, stimulating the migration of key pro-inflammatory cells (Th17 cells) to the sites of CNS damage [128]. In MS, astrogliosis or astrocyte injury may be the result of neuroinflammation [129] and IL-1 and IL-6 appear to be the significant intermediaries. A tightly packed astrogliosis defines persistent focal lesions in MS, while other factors may contribute to astroglial damage including astrocytic *β*2 adrenergic receptor insufficiency, overexpression of endothelin 1, and tissue transglutaminase [297]. In vivo inhibition of astrogliosis or reactive astrocytes revealed that astrogliosis is required for proper CNS regeneration, neuronal defence, BBB safeguards, and inflammation resolution [298]. Overall, reactive astrocytes in MS lesions have both positive and negative effects on neuroinflammatory disorders, depending on the conditions and variability of astrocytic aggregates [129]. Chemo-attractive or chemo-repulsive elements may allow the entry of damaging immune cells while simultaneously assisting in the entrance of oligodendrocyte progenitors, which are necessary for regeneration. Pro-inflammatory factors may kill normal oligodendrocytes, myelin, and axons while attacking pathogenic cells; defensive trophic agents may also breach the BBB, alter the extracellular matrix, and allow CNS-specific immune cells to infiltrate and remain following tissue injury, or a prolonged glial scar may provide physical integrity, restrict future harmful exposures, and prevent the entry of compensatory cellular components into the injured area [299].

## 15. Eosinophils and Basophils

According to recent neuroimmunology research, eosinophils are essential for the signalization of CSF by inflammatory disorders of the CNS and its leptomeningeal coverings [300]. Similarly, basophils are essential and nonredundant in the regulation of type 2 immunity, resistance to parasite infection, autoimmunity, and autoimmune diseases. They share phenotypic and functional characteristics with MCs, which have significant protective benefits after spinal cord damage and traumatic brain injury [301], despite being one of the most pro-inflammatory cell types in the body. To date, little is known of the potential significance of basophils or eosinophils in MS. An increased number of basophils have been reported in MS compared to healthy individuals [302], and while eosinophil numbers and EOTAXIN-1 concentration were notably higher following MOG_35–55_-induced EAE in C57BL/6 mice when ablated in a mouse model, there was no effect on neuroinflammation, demyelination nor clinical progression, indicating that they were not required for disease progression [302, 303].

## 16. Contribution of Subsets of Innate-Like T and B Lymphocytes in MS

Innate-like T lymphocytes are a type of T cell that functions as a link between the innate and adaptive arms of the immune system, using TCRs to detect exterior ligands yet also displaying innate-like characteristics. These cells may play a key role in the progression of MS [1] with some displaying important roles in CNS inflammation as mediators, while others may have immunomodulatory activities in pathology. Gamma delta T (*γδ*T) cells, NKT cells, and mucosal-associated invariant T (MAIT) cells, all of which belong to this family of lymphocytes, have shown an association with the pathogenesis of MS and EAE, while the role of innate-like B (ILB) cells in CNS autoimmunity has only recently been assessed [143]. In addition, a number of studies that link innate lymphoid cells (ILCs), in particular ILC 1-3s, to the pathophysiology of MS have raised awareness of the significance of ILCs [141].

## 17. *γδ* T Cells

*γδ* T cells are a diverse range of lymphocytes with the capacity to produce chemokines, cytokines, and inflammatory and cytotoxic mediators to augment inflammatory reactions, modulating differentiation/apoptosis of damaged cells. They thus have the capacity to promote autoimmune functions in MS [304] if they access the CNS, causing harm to oligodendrocytes and contributing to CNS demyelination and fibrosis [130]. *γδ* T cells have been identified in greater numbers in MS white matter plaques and cerebral fluid than in peripheral blood [131] and they colocalize with HSP65^+^OGC and are oligoclonally confined to V*δ*2J*δ*3 lineages. Human adult-derived oligodendrocytes can be lysed in vitro by *γδ* T cells [132]. HSP-70 was also shown to react to a subset of *γδ* T lymphocytes in MS abnormalities, while non-CNS-specific antigens were identified as playing a role in the underlying pathology [133, 305, 306]. The *γδ* TCR repertoire is altered in MS CSF, suggesting that *γδ* T cells responding to brain-derived antigens may have been locally increased [307].

The pathogenic role in EAE of *γδ* T cells is via control of migration of inflammatory cells into the spinal cord and boosting of their pro-inflammatory cytokine content [308]. Excited *αβ* T cells can trigger *γδ* T cells, and these interactions are increased in MS after vaccination using MBP-sensitive T lymphocytes, indicating that *γδ* T cells have a role in marginal processes that regulate stimulated autoimmune T cells [309] resulting in myelin and oligodendrocyte degeneration [310].

Other *γδ* T cell subsets appear to contribute to MS pathogenesis and prevention, serving several roles in CNS inflammation as well as demyelination. The V*γ*4^+^ subset generates a variety of pro-inflammatory cytokines, particularly IL-17, representing 15%–20% of IL-17-producing cells in the CNS, although with a different transcriptional program than CD4^+^ Th17 cells. The V*γ*1^+^ subset, on the other hand, releases CCR5 ligands, which may aim at regulatory T cell differentiation [311]. Moreover, in RRMS, V*δ*1^+^*γδ* T cells display the platelet endothelial cell adhesion molecule 1 (PECAM1; CD31) which facilitates its transendothelial trafficking ability. In contrast, the V*δ*2^+^ subset occurs in greater numbers and migrates via the NK receptor protein 1a (KKRP1a; CD161) signaling pathway, mediated by IL-12. CD16 is a minimal Fc*γ* receptor, *γδ* T cell activator, and a cytotoxicity mediator. In MS patients, the frequency of CD16^+^*γδ* T cells is higher in response to inflammatory cytokines including IL-2 and IL-15, which have been linked to disease progression [312]. In addition, CD161^high^CCR6^+^*γδ* T cells release IL-17 that may play a part in the localized inflammatory reaction in the CNS of MS subjects given that its distribution in the CSF of people with CIS/MS in relapse is considerably greater [313].

Considerably fewer total *γδ* and *γδ*2 T cells have been found in people with MS compared to a healthy control group, possibly indicative of either *γδ*2 T cell apoptosis or CNS translocation [314]. Through Fas/FasL-induced apoptosis of encephalitogenic T cells, *γδ* T cells control both inflammation in the CNS and disease repair, and a prompt resolution of inflammation is critical to avoid the development of irreversible damage to the CNS in chronic illness [315].

## 18. Natural Killer T (NKT) Cells

NKT cells are innate immune effectors exhibiting both T and NK cell receptors (TCR and NKR) and recognize glycolipid antigens in the context of CD1d. Although they represent a small percentage of lymphocytes, they have profound immunomodulatory roles in a variety of diseases, as they exhibit characteristics of both innate and adaptive immune responses having cytotoxic and immunoregulatory abilities [316]. They have been broadly divided into two subpopulations, type I and type II based on their TCR usage and lipid antigen specificity. Given that the brain contains several glycophospholipids, it is not surprising that a role for NKT cells has been reported in some neurological diseases, including MS, although evidence at the present time is still limited. The quantification of T cell populations conveying the NKR CD56, CD161, and CD94 in peripheral blood of MS patients revealed that CD161^+^ T cells and CD94^+^ T cells were dramatically reduced in PPMS and SPMS individuals, while CD56^+^ T cells remained relatively stable in number [317]. During EAE, large subsets of type II NKT cells are specifically abundant in CNS tissue and identify as myelin-derived sulfatides. Activation of type II NKT cells by the addition of sulfatide inhibited induction of T cell-mediated autoimmune disease by repressing inflammatory class II MHC-restricted pathogenic CD4^+^ T cell reactions [318]. It is postulated that NKT cells may proliferate in MS patients' CSF as a response to the disease's immunological reactivity in the CNS [115].

Invariant NKT (iNKT) cells [319] (V*α*24-J*α*18, V*β*11-restricted in humans; V*α*14-J*α*18, V*β*8.1, 2 in mice) were found to be elevated in MS patients [317]. iNKT cells are an important subset of NKT cells and can rapidly produce cytokines following an initial stimulus, important for Th cell polarization [320]. They have recently been classified depending on the cytokine and transcription factors they express, Th1-like (iNKT1); Th2-like (iNKT2), Th17-like (iNKT17), and Tfh-like (iNKTfh) iNKT cells. Moreover, they play an immunoregulatory role in autoimmunity by activating immune cells and inducing cytolytic activity [321]. Through pattern recognition receptors, native self-antigens, microbial antigens, and possibly other hazardous antigens can activate iNKT cells to become essential mediators of regulatory Th17 or pro-inflammatory Th1 cytokine responses, in the presence of cytokines IL-10, IL-12, or IL-17 [319]. We and others have shown that increasing NKT cells in EAE-induced B6 mice had a protective effect on disease. However, different mouse backgrounds or different EAE models tend to show contradictory results, so this requires further investigation.

## 19. Mucosal-Associated Invariant T (MAIT) Cells

MAIT cells are defined by their expression of a semi-invariant *αβ* TCR which recognizes biosynthetic derivatives of riboflavin synthesis presented on MR1. Studies of MS subtypes and EAE have been inconsistent as to whether MAIT cells have a role in or provide a beneficial or detrimental role in MS pathology [1]. The number of MAIT cells in the blood, as well as ex vivo activation capacity and effector phenotype as evidenced by transmigration ability in an in vitro BBB model, was equivalent between RRMS patients and control subjects. However, there was transcriptional upregulation of MRI, and in MS pathogenic stimulating cytokines IL-18 and IL-23 in MS lesions, possibly indicating a minor role due to the small number of infiltrating MAIT cells observed [135]. Numerous MAIT cells can however produce IL-17, resulting in increased proliferation of MAIT cells in MS patients, which together with IL-7R upregulation suggests that these unusual T cells play an aggressive role in MS pathogenicity [136].

A specific depletion of circulating CD8^+^CD161^high^ T cells, which are mostly MAIT cells, was first described in PPMS patients [322] and later in RRMS patients, where cells that were CD3^+^TCR*γδ*-V*α*7.2^+^CD161^high^ were greatly diminished along with pro-inflammatory cytokines, especially at exacerbation of disease. The proportion of MAIT cells in the CSF of MS patients was considerably greater than in peripheral blood in paired samples, indicating that MAIT cells can cross the BBB. Only limited TCRV*β* repertoires persisted throughout time [323] and Tc17-like MAIT cells were negatively associated with CNS myelin degeneration. They were decreased in blood from individuals with PPMS, detected by flow cytometry study of circulating MAIT cells and MAIT cell subsets presenting CXCR3 and CCR6, implying that they have deleterious acquisition functions in MS, and are also a link to CNS lesion [137].

## 20. Innate-Like B (ILB) Cells

By generating a prompt T cell-independent antibody response, ILB cell subtypes serve as a link between the fast-acting innate and the slower acting initial T cell-dependent adaptive antibody response. ILB cells are a mixed population of atypical B cells that are sensitive to foreign substances and can defend against a variety of illnesses but also respond to self-antigens [138]. They are made up of B1 cells, marginal zone (MZ) B cells, and other associated B cells in mice [324] and a putative human homologue, CD20^+^CD27^+^CD43^+^, have also been identified [325]. B1 cells contribute to antibody production and act as APCs, but they have no memory and are not part of the adaptive immune response. B1 and MZB cells in mice are seen to play a key role in autoimmunity mitigation by producing regulatory cytokines (IL-10) and natural antibodies (IgM) although the mechanisms involved are not well established in MS [326].

In a normal brain, B1 cells are basic cells that produce natural antibodies and anti-inflammatory chemicals and may exert a more major role in MS pathophysiology than previously supposed [139]. The proportion of the CD27^+^CD43^+^ B1 cell subpopulation was reported as dramatically reduced in RRMS patients [327] with the number of B1 cells being negatively correlated to the period from the preceding relapse.

B cells and CD20^+^ T cells were effectively suppressed by the MS immunomodulator, ofatumumab, while MZB cells in the spleen and LNs appeared to be conserved [328]. Treatment with natalizumab, on the other hand, resulted in a higher level of MZ-like B cells [329]. Lower expression of CD32b (Fc*γ*RIIb), an inhibitory receptor of B cells, was identified on IgM^+^ B cell subsets in females with MS or CIS compared to normal healthy individuals and was associated with anti-EBV viral capsid antigen IgM antibodies and increased B cell stimulating factor serum levels. However, no consequence of reduced expression was detected even though in vitro polyclonal activation of B cells in the presence of IgG immune complexes, which resulted in reduced TNF, could be reversed in normal healthy control cells by the addition of a CD32b blocking antibody. This suggests that naive and MZ-like B cells may play a role in the immunopathogenesis of MS, at least in female patients [330].

## 21. Innate Lymphoid Cells (ILC)

ILCs straddle both arms of the immune system, as they respond quickly to infection and secrete a suite of inflammatory mediators similar to T lymphocytes but without expression of antigen receptors, or the ability to undergo clonal selection and expansion when stimulated [331]. They control both acute and chronic inflammation. ILC precursors may migrate to infected or injured tissues, from their primary site of production, where they undergo cytokine-dependant maturation or mature in response to stress ligands or bacterial or dietary compounds They are divided into five distinct subsets based on phenotypic and functional profiles. Group 1 ILCs are made up of classical NK cells (considered counterparts of cytotoxic CD8^+^ T cells) and ILC1 cells that produce IFN-*γ* (representative of a Th1 counterpart); Group 2 ILCs are made up of ILC2 cells that produce IL-4, IL-5, and IL-13 (a counterpart of Th2 cells); and Group 3 ILCs are made up of ILC3 cells that produce IL-17 and IL-22 (counterpart of Th17 cells) along with LTi cells, which are believed to be involved in the development of secondary lymphoid organs. ILCs induce innate responses in stromal, epithelial, and myeloid cells, dependant on which cytokines are produced. They also activate tissue-resident DCs to migrate to LNs to elicit specific T cell responses (which also regulate the ILC) and can directly regulate T cells via the presentation of peptide antigen on MHCII. ICLs are also involved in immunopathology and recent research has demonstrated that ILC responses are modulated in patients with MS following treatment with the CD25-specific monoclonal antibody, Daclizumab [253]. The exact role of ILCs in MS, however, is unknown as contradictory studies have indicated that they may either accelerate or protect against disease as they release pro-inflammatory cytokines that exacerbate the immune response in MS, as well as cytokines that regulate T-cell inflammation [141]. ILC subtypes can enter the CNS and stay in the meninges, according to recent research in EAE. Although more research is required, the increased quantity and activation of LTi cells in CNS meninges during EAE suggested that these cells support the pathogenesis of EAE [142] but as ILC display plasticity, converting from one form to another depending on their microenvironment, determining their precise mechanism and function is currently a challenge.

## 22. Discussion and Concluding Remarks

The intricacy of MS, as with other autoimmune diseases, is becoming ever more apparent. Not only is there the complex interaction between genes, the epigenome and the environment, but there is the added complication of misdiagnosis and the convoluted interaction of innate and adaptive immune cells of the immune system: how precisely does each cell interact with other innate, adaptive, and nonhaematopoetic cells, do these contribute to promoting, resolving, or constraining inflammation, and is the inflammation itself detrimental or involved in disease resolution? Demyelination, remyelination, and the introduction of new neural network connections and the plasticity of cells, as they adapt to the ever-changing environment, make understanding the underlying mechanisms difficult to elucidate. While research indicates the involvement of many immune subtypes in the deleterious effects of disease onset and progression, there are also many indications for protective or opposing roles. The recent discovery of the glymphatic system and meningeal lymphatics has helped in our understanding of how peripheral immune cells may enter the brain to enable communication with resident cells, but clinical presentation and experimental interpretation are being hampered by the very nature of the disease: de- and re-myelination, new neural connections, the plasticity of immune cells, and pleiotropic characteristic of immune mediators, together with the circadian rhythm of gene expression and cytokine levels, and interaction of other immune modulators/hormones, as to whether we are examining initiators or effectors of pathogenesis or the consequences of timing or inflammation.

Most current therapeutics, that are immunomodulating, are based on CD4^+^ T cells, especially Th1 and Th17, or are monoclonal antibodies (Table 1), and although some therapeutics are successful, there is no one-size-fits-all and several side effects are present. There are now numerous indicators to suggest that the development of successful treatments will require modifications to some other immune cell subsets and perhaps combination therapies will target the disease more effectively. There is currently, little to no information available regarding certain immune cell types and the list is ever-growing as new markers of definition are discovered, for us to achieve a better understanding of the fundamental mechanisms of MS, more refined cell analyses and their interactions are necessary. In addition, functional/translational studies must be critically evaluated, with special attention to conclusive MS diagnoses, medication use, time of day tissue collection, ensuring healthy and disease-free control tissues, longitudinal samples, having standardized protocols, and having a thorough consideration of interactions caused by genetic and environmental variables. The drivers of underlying networks leading to disease pathology should soon be realized with the development of ever more potent techniques such as scRNA sequencing in conjunction with spatial proteomics. This will also provide a strong base for identifying potential therapeutic candidates to impede MS progression and eradicate it in the future, and we can forever “kiss goodbye to MS.”

## Figures and Tables

**Table 1:** Current MS treatments, including mode of action and potential side effects (as described by MS Australia).

Treatment	Mode of action	Potential side effects
*Injectable DMTs*		
*β* interferon drugs (i) Avonex, rebif (interferon *β*-1a) (ii) Betaseron, extavia (interferon *β*-1b) (iii) Plegridy (peginterferon *β*-1a)	Regulation of immune system by suppressing expression of pro-inflammatory cytokines, promoting the expression of anti-inflammatory cytokines, and preventing inflammatory cells from entering the CNS	Serious: liver failure; mental health problems (including depression or suicidal thoughts); heart and blood vessel problems, seizures; and skin necrosis. Common: pain, swelling, flu-like symptoms; low white blood cell count (Betseron, Plegridy)
Copaxone, glatopa (glatiramer acetate)	Block immune system attack of myelin sheath, by shifting the balance between pro-inflammatory and Treg cells, and inhibits APCs by competing for myelin antigens	Serious skin necrosis, lipoatrophy, and liver failure. Common: pain/swelling/redness at injection site, flushing, trouble breathing, rash, and chest pain
Kesimpta (ofatumunab)	Selectively depletes CD20-expressing B cells	Hepatitis B Virus (HBV) reactivation can occur, in some cases resulting in fulminant hepatitis, hepatic failure, and death. Progressive multifocal leukoencephalopathy (PML) resulting in death. Neutropoenia, anaemia, thrombocytopenia. Common: Back pain, muscle aches, redness of the skin, and trouble sleeping
*Oral DMTs*		
Gilenya (fingolimod)	Sphingosine 1-phosphate receptor modulators. This drug is believed to work by trapping T cells in the lymph nodes, preventing them from entering the brain and spinal cord	Serious: liver damage, severe worsening of MS following drug withdrawal, progressive multifocal leukoencephalopathy (PML), basal cell carcinoma, and melanoma. Macular edema, posterior reversible encephalopathy syndrome (PRES). Common: can slow the heartbeat, esp. initially
Mayzent (siponimod) and zeposia (ozanimod)	Similar to Gilenya (they modulate S1PR1 and S1PR5 on lymphocytes)	Serious: life-threatening infections, macular edema, PRES, liver, and breathing problems, severe worsening of MS after stopping the drug, basal cell carcinoma, and melanoma. Common: can slow the heartbeat, esp. initially headache, high blood pressure, and abnormal liver tests (common with Mayzent), upper respiratory tract infections, increased liver enzymes, back pain, orthostatic hypotension, headache, high BP, painful, and frequent urination (zeposia)
Tecfidera (dimethyl fumarate)	Anti-inflammatory and antioxidant properties (via activation of NRF2 and inhibition of GAPDH) that help protect against damage to the brain and spinal cord	Serious: allergic reactions, PML, a lowering of your white blood cell count, liver problems, herpes zoster infection (shingles), and other serious infections. Common: flushing, redness, itching, rash, nausea, vomiting, diarrhea, stomach pain, and indigestion
Vumerity (diroximel fumarate)	Similar to tecfidera but has been found to be better tolerated with fewer gastrointestinal side effects	Serious: allergic reactions, PML, a lowering of your white blood cell count, liver problems, shingles, and other serious infections. Common: flushing, redness, itching, or rash and stomach problems, including nausea, vomiting, diarrhea, pain, or indigestion
Bafiertam (monomethyl fumarate)	Similar to tecfidera and vumerity but improved gastrointestinal tolerability	As for vumerity (diroximel fumarate)
Aubagio (teriflunomide)	Blocks mitochondrial respiration by inhibiting dihydro-orotate dehydrogenase, thereby reducing activated T and B cell proliferation	Serious: low white blood cell count, more frequent infections, numbness and tingling in the hands or feet, high blood pressure, allergic reactions, potentially life-threatening skin reactions, new or worsening breathing problems. Common: headache, diarrhea, nausea, hair thinning or loss (alopecia), and abnormal liver blood tests
Mavenclad (cladribine) (generally only recommended for patients who do not tolerate or respond to other DMTs)	Temporarily reduces the number of T and B lymphocytes by impairing DNA synthesis/repair and inducing cell death (is a deaminase-resistant adenosine analog)	Serious: low blood cell counts, increased risk of cancer, serious infections (e.g., tuberculosis, hepatitis B or C, shingles, or PML), liver problems, allergic reactions. Common: upper respiratory infections, headache, and low white blood cell counts
Ponvory (ponesimod)	S1R1 on lymphocytes is selectively modulated to prevent their exit from lymphatic tissue	Serious: upper respiratory infection, increased hepatic transaminase and hypertension, basal cell carcinoma, melanoma, squamous cell carcinoma, macular edema. Common: dyspepsia, urinary tract infection, hypercholesterolemia, dizziness, and migraine
*Infused DMTs*		
Lemtrada (alemtuzumab) (Due to safety concerns, the FDA recommends this drug be reserved for patients who do not respond to two or more other DMTs)	A monoclonal antibody that targets lymphocytes via CD52. (Can also deplete some subsets of myeloid cells)	Serious: immune thrombocytopenic purpura (ITP) in which the body attacks the platelets, and kidney problems, serious infusion reactions, stroke, and tears in carotid and vertebral arteries, increased risk of certain cancers. Hyper- or hypothyroidism, low blood cell counts, liver inflammation, hemophagocytic lymphohistiocytosis, thrombotic thrombocytopenic purpura (TTP), bleeding disorders, serious infections (e.g., PML, tuberculosis, listeria), acalculous cholecystitis, and pneumonitis. Common: skin rashes, swelling of nose and throat, nausea, vomiting, diarrhea, musculoskeletal and neurologic pain, and thyroid problems
Novantrone (mitoxantrone)	Antineoplastics (anticancer/chemotherapy). It reduces the activity of T cells, B cells, and macrophages (through DNA intercalation leading to cross-links and strand breaks) to prevent them from attacking the myelin	Serious: AML, heart toxicity specifically heart failure. Common: nausea, hair loss, menstrual changes, upper respiratory, and urinary tract infections, mouth sores, irregular heartbeat, diarrhea/constipation, back pain, headache, and blue–green urine
Tysabri (natalizumab)	A monoclonal antibody that blocks VLA4, preventing potentially harmful immune system cells from crossing the BBB into the CNS	Serious: PML, herpes infections, liver damage, allergic reactions, weakened immune system and higher risk of infections, and low platelet counts. Common: headache, nausea, diarrhea, urinary tract/lung/nose and throat infections, depression, extreme tiredness, joint pain, vaginitis, and rashes
Ocrevus (ocrelizumab)	A monoclonal antibody that targets CD20-positive B lymphocytes, depleting them	Serious: severe infusion reactions, infections (e.g., herpes viral, PML, and reactivation of hepatitis B), weakened immune system, and risk of cancer, including breast cancer. Common: upper respiratory tract infections and infusion reactions in RRMS patients; upper/lower respiratory tract infections, skin infections, and infusion reactions (PPMS patients)
Briumvi (ublituximab)	A monoclonal antibody that targets CD20-positive lymphocytes	Serious: severe infusion reactions and infections (e.g., herpes virus, PML, and reactivation of hepatitis B) Common: upper and lower respiratory tract infections, infusion reactions, and herpes infections
Rituxin ^*∗*^ or MabThera (rituximab) Administered under close supervision of physician	A monoclonal antibody that targets CD20-positive lymphocytes (B cells)	Serious: immunoglobulin deficiency syndromes can occur that may be associated with severe infections, progressive multifocal leucoencephalopathy (PML), a rare but serious brain infection caused by a virus, can occur in people with prior or having concurrent immunosuppressive therapy, although the chances are listed as “no or very low risk,” hypotension, hypertension, arrhythmias, and angina. Common infusion-related reactions, esp. after the initial dose

^*∗*^Not officially used for treatment of MS but is administered off-label by several neurologists for relapsing forms of MS. It is approved for treatment of adults with B cell non-Hodgkin's lymphoma, chronic lymphocytic leukaemia, rheumatoid arthritis, granulomatosis with polyangiitis (Wegener's granulomatosis), microscopic polyarteritis, and pemphigus vulgaris.

**Table 2:** Immune cell type and site of action in MS.

Cell type	Action site	References
CD4 T cells	Activated myelin-reactive CD4^+^ T cells are present in the blood and cerebrospinal fluid (CSF) of MS patients. Found predominating in acute lesions (perivascular infiltrates)	[47, 59–66]
B cells	Located in the meninges and in the large perivascular spaces around the cerebral ventricles. While CD20^+^ B lymphocytes predominate and may have pro-inflammatory properties in early and active lesions, the number of plasma cells with potential anti-inflammatory properties increases in later stages	[67–80]
Macrophages	Major effectors of inflammation and demyelination in both MS and EAE found in MS lesions	[7, 48, 58, 81–89]
Myeloid	Aberrant myeloid function is often observed in MS plaques in the CNS	[48, 90, 91]
Microglia/macrophages	Shift in M1/M2 balance is important for disease pregression. Early phase: immediate activation to become classically activated macrophages (M1 cells), releasing pro-inflammatory cytokines and damaging CNS tissue. Later phase, microglia/macrophages in the inflamed CNS are less activated, present as alternatively activated macrophage phenotype (M2 cells), releasing anti-inflammatory cytokines, accompanied by inflammation resolution and tissue repair	[58, 83, 85, 86, 92–96]
CD8 T cells	Frequently found in chronic lesions (perivascular infiltrates)	[48, 63, 66]
Tregs	In CSF, they appear to be defective and dysregulation of suppressive and migratory markers on Tregs into CNS has been linked to MS	[97–100]
Dendritic cells (DCs)	Found to be increased in the brain parenchyma and myelin-reactive T cells require the involvement of CNS microvessel-associated DCs to recognize the local antigen	[101–105]
Neutrophils	In MS patients, neutrophils have been identified in the cerebral fluid, and also postmortem CNS material demonstrated neutrophil infiltration in areas where the BBB was leaking	[106–112]
Natural killer cells	NK cells are seen in the CSF of MS patients and despite having a low absolute number, they are highly active cytotoxin molecules that can kill autologous activated immune cells as well as other stressed cells	[113–118]
Mast cells	Mast cells (MCs), which produce histamine and are present before any pathological or clinical symptoms of MS, are found in the thalamus and hypothalamus of the brain	[119–121]
Astrocytes	Astrocytes become active in demyelinating lesions. They had noticeably higher levels of natural cytotoxicity triggering receptor 1 (NCR1) expression in MS white matter lesions. During demyelination, astrocytes generate significant quantities of matrix metalloproteinases (MMPs), that aid tissue damage and reorganization, and produce gliotic scars in inactive lesions and help maintain the BBB and the blood-spinal cord barrier, as well as remyelination and neuronal integrity	[122–129]
*γδ* T cells	*γδ* T cells have been identified in greater numbers in MS white matter plaques and cerebral fluid, causing harm to oligodendrocytes and contributing to CNS demyelination and fibrosis	[130–133]
Natural killer T (NKT) cells	NKT cells may proliferate in MS patients' CSF as a response to the disease's immunological reactivity in the CNS	[115, 134]
Mucosal-associated invariant T (MAIT) cells	The proportion of MAIT cells in the CSF of MS patients was considerably greater than in peripheral blood in paired samples, indicating that MAIT cells can cross the BBB	[135–138]
Innate-like B (ILB) cells	CD5^+^ (innate) B cells are a provider of persistent antilipid IgM antibodies, which can be observed in the CSF as a portion of the oligoclonal IgM bands	[139, 140]
Innate lymphoid cells (ILC)	In EAE, ILC subtypes can invade the CNS and remain in the meninges. They release cytokines that control T-cell inflammation as well as pro-inflammatory cytokines that worsen the immune response in MS	[141, 142]

## Data Availability

No underlying data were collected or produced in this study. Prior studies (and datasets) referred to within the manuscript are cited at the relevant places within the text as references. Figure 1 was created with BioRender (https://BioRender.com), and the current treatments included in Table 1 have been gleaned from information supplied by MS Australia (https://www.msaustralia.org.au) and Therapeutic Goods Administration package insert information.
